# Supplementary material for: Inadequate lumbar protection with load weight limits based on body weight percentages: An experimental and simulation study of the weight limits set by the Japanese guidelines for preventing low back pain
Source: PLoS One. 2025 Jun 25;20(6):e0327175. doi: 10.1371/journal.pone.0327175 (PMC12193010; doi:10.1371/journal.pone.0327175)
Supplement: Attachment — Submitted filename: R1response.docx [file pone.0327175.s004.docx]

Academic Editor:

Thank you for considering our submission. Below are point-by-point responses to the issues that were raised.

1. The presentation of results needs to be clearer, particularly by adding summary tables.

- We have added a summary table (Table 4) to enhance the clarity of the presented results.

2. The discussion section should go beyond simply restating the results. It needs to offer more analytical depth and clearer implications for guideline development.

- We have reorganized the Discussion section and included a description of future guideline development incorporating the experimental findings.

3. The conclusion currently does not provide critical reflection and needs improved articulation of the significance of the study, suggesting possible future directions.

- We have revised the Conclusion section to clearly state the key findings that address the objectives of this study. Additionally, we have added a description of future directions.

4. The authors should emphasise the central finding that weight limits based solely on body weight percentages do not adequately prevent lumbar strain.

- We have revised the Conclusion section and emphasized the key finding that load weight limits based on body weight percentages are inadequate for lumbar protection.

Reviewer #1:

Thank you for reviewing our manuscript. Below are the responses to the comments.

1. Lines 45-47: the values given refer to the best conditions for manual handling of loads. It is appropriate to specify this in the text.

- We have revised the description of the International Organization for Standardization (ISO) guidelines and clarified that the values are “the maximum recommended load weight under optimal conditions” (Lines 44–45).

2. Lines 56-60: perhaps the frequency of movements and the duration of the tasks are not considered either? If so, it is appropriate to specify this.

- The Ministry of Health, Labor and Welfare of Japan (MHLW) guidelines do not set load weight limits based on load handling positions, frequency of movements, or duration of tasks. We have revised the description of the MHLW guidelines (Lines 57–60).

3. Lines 102-103: it is necessary to add more information regarding the ease of gripping the load: were there handles or not? It would be useful to add a photo or a figure.

- As mentioned in lines 102–103, the load was a container with handles on both sides. We have added Fig 2, which shows the container and metal weights.

4. Lines 330-331: the frequency of movements and the duration of the task were not considered either (factors that certainly influence the risk).

- We have clarified that the frequency of movements and duration of the task were not considered for the weight limits and included these factors in the paragraph where the shortcomings of MHLW guidelines are discussed (Lines 310–313).

Reviewer #2:

Thank you for reviewing our manuscript. Below are the responses to the comments.

1. Methods:
   In general a graphical representation of the various positions to be performed by the participants could help the understanding of the experimental method that was done. As well as a graphical description of the 3d motion analysis could be useful.

- We have added Fig 3, which shows various positions performed by the participants and examples of the captured motion data (Lines 125–128).

2. Results:
   lines 223-268: a summary table could improve the understanding of the results.

- We have added ANOVA summary tables (Table 4) and removed the numerical description of the ANOVA results from the main text (Lines 222–264).

3. Discussion:
   lines 271-300: this whole part seems like a repetition of the results.
   Give more support for the design of new guidelines that really prevent the problem of low back pain.

- We have removed this part and provided more support for the future development of new guidelines (Lines 314–342).

4. Conclusion:
   The conclusions lack critical sense of the work and future developments are absent.

- We have highlighted the valuable insights gained from this study in the first paragraph of the Conclusion section (Lines 356–363). Additionally, we have included descriptions for future development in the second paragraph (Lines 364–369).
