# Supplementary material for: Inadequate lumbar protection with load weight limits based on body weight percentages: An experimental and simulation study of the weight limits set by the Japanese guidelines for preventing low back pain
Source: PLoS One. 2025 Jun 25;20(6):e0327175. doi: 10.1371/journal.pone.0327175 (PMC12193010; doi:10.1371/journal.pone.0327175)
Supplement: Attachment — Submitted filename: R2response.docx [file pone.0327175.s005.docx]

Academic Editor:

Thank you for considering our submission. Below are the responses to the issues that were raised.

1. That said, I believe the manuscript would benefit from a more thorough treatment of its methodological and contextual limitations. Specifically, I suggest adding a clearly labeled “Limitations” section (or expanding the existing discussion) to address the following points:
   - The use of uniform motion capture data across simulated body weights, which may not reflect actual kinematic variations.
   - The omission of task duration and frequency—factors known to influence low back pain risk.
   - The application of a fixed 3400 N IVD-CF threshold, despite known individual variability in lumbar spine tolerance.
   - The limited generalizability of the findings beyond the Japanese working population.
   - The practical challenges of implementing alternative guideline methods, and the absence of a proposed framework tailored to Japan.

Acknowledging these issues directly will enhance the interpretability, rigor, and credibility of your study. I believe these additions will strengthen the manuscript and support its value to both researchers and policymakers.

- In response to your comments, we have added the relevant limitations to the Discussion section.

Journal Requirements:

1. Please review your reference list to ensure that it is complete and correct. If you have cited papers that have been retracted, please include the rationale for doing so in the manuscript text, or remove these references and replace them with relevant current references. Any changes to the reference list should be mentioned in the rebuttal letter that accompanies your revised manuscript. If you need to cite a retracted article, indicate the article’s retracted status in the References list and also include a citation and full reference for the retraction notice.

- We appreciate your bringing the inaccuracies in our reference list to our attention. In response, we have conducted a thorough review to ensure that all references are accurate and complete. We have also verified that no retracted publications are included. Furthermore, we have made the following revisions to the citations:

[3]:	Replaced the URL to directly link to the cited document file.

[5]:	Corrected the country name from “England” to “United Kingdom”.

[6]:	Replaced the citation with the book titled “2023 TLVs and BEIs”.

[19]:	Added “(in Japanese)” to the end of the title, replaced the URL to directly link to the cited document file, and included the access date.
